# N-Acetylcysteine Ineffective in Alleviating Hangover from Binge Drinking: A Clinical Study

**DOI:** 10.3390/toxics12080585

**Published:** 2024-08-12

**Authors:** Boris Podobnik, Lenart Demšar, Lucija Šarc, Aleš Jerin, Joško Osredkar, Jurij Trontelj, Robert Roškar, Miran Brvar

**Affiliations:** 1 Centre for Clinical Toxicology and Pharmacology, University Medical Centre Ljubljana, 1000 Ljubljana, Slovenialenart.demsar@gmail.com (L.D.); lucija.sarc@kclj.si (L.Š.); 2 Faculty of Medicine, University of Ljubljana, 1000 Ljubljana, Slovenia; 3 Institute of Clinical Chemistry and Biochemistry, University Medical Centre Ljubljana, 1000 Ljubljana, Sloveniajosko.osredkar@kclj.si (J.O.); 4 Department of Clinical Biochemistry, Faculty of Pharmacy, University of Ljubljana, 1000 Ljubljana, Slovenia; 5 Department of Biopharmaceutics and Pharmacokinetics, Faculty of Pharmacy, University of Ljubljana, 1000 Ljubljana, Slovenia; jurij.trontelj@ffa.uni-lj.si (J.T.); robert.roskar@ffa.uni-lj.si (R.R.)

**Keywords:** alcohol, hangover, veisalgia, NAC, sodium, dehydration, oxidative markers

## Abstract

Alcohol hangover (veisalgia) is a fairly common phenomenon. The pathogenesis of veisalgia is not understood and treatment has not yet been established. Occasionally, students take N-acetylcysteine (NAC) before binge drinking to alleviate hangover. The aim of this study was to evaluate the effect of NAC on serum levels of electrolytes, enzymes, oxidative stress biomarkers and symptoms of veisalgia in binge drinking. In this randomized, double-blind, placebo-controlled study, healthy students were randomly assigned into two groups: one receiving NAC and the other receiving a placebo. Blood samples were taken before drinking, 30 min after a 1.5 h long drinking session, and the subsequent morning. Serum levels of electrolytes, urea, enzymes, ethanol, 8-Hydroxydeoxyguanosine (8-OHdG) and N-epsilon-hexanoyl-lysine were measured. The participants completed the Acute Hangover Severity Scale (AHSS) assessment based on symptoms, and 40 students (20 male), aged 23 ± 2 years, were included in the study. Their mean blood ethanol concentration was 1.4 g/kg. Serum sodium levels were increased after drinking, and urea decreased the following morning compared to their levels before drinking in both groups. Serum 8-OHdG levels were increased after drinking and remained elevated until the following morning, compared to the levels before drinking, in both groups. NAC had no effect on sodium, urea and 8-OHdG levels or the symptoms of veisalgia. In conclusion, binge drinking causes a transient increase in serum sodium and as a prolonged increase in oxidative marker 8-OHdG levels. NAC had no effect on the sodium and 8-OHdG levels.

## 1. Introduction

An alcohol hangover (veisalgia) refers to the combination of negative mental and physical symptoms that can be experienced after a single episode of alcohol consumption, starting when blood alcohol concentration (BAC) approaches zero. Hangovers are characterized by a mix of symptoms affecting mood, cognition, and physical functioning. These symptoms, which can persist for up to 24 h, have been shown to negatively impact daily activities, including job performance and driving [1,2].

Despite being a relatively common occurrence, the pathogenesis of veisalgia is still not well understood, and there is currently no established treatment. The pathogenesis of veisalgia may involve various factors, including ethanol, dehydration, hypoglycemia, inflammation, oxidative stress, the presence of biological active compounds in alcoholic beverages (such as congeners), hormonal fluctuations, changes to the immune system and individual genetic factors [3,4].

Alcohol hangover has detrimental effects not only on the physical health, psychological well-being, and social life of an individual but also on the economy of a country. This highlights the need to discover an effective treatment and/or preventive strategy for alcohol hangover [1].

Unfortunately, there is only very low-quality evidence of the efficacy of any pharmacological intervention for the treatment or prevention of alcohol-induced hangover in the scientific literature. The most promising interventions include clove extract, tolfenamic acid, and pyritinol [3]. On the other hand, there are many anecdotal reports of efficacious therapies. One such therapy is N-acetylcysteine (NAC), which is very popular among medical students in Ljubljana, Slovenia. Students often take NAC before binge drinking to alleviate hangover. However, NAC is also used to alleviate alcohol hangovers in other countries, such as the USA [5].

As an antioxidant that regenerates intracellular glutathione, NAC could interfere with the pathogenesis of veisalgia by reducing the oxidative stress caused by alcohol metabolism. Additionally, NAC could lower the levels of acetaldehyde, one of the toxic metabolites of alcohol, by forming adducts [6,7,8,9].

In a recent study investigating the effect of NAC on mitigating hangover symptoms, no significant difference between the NAC and placebo groups was found [10]. However, there was a statistically significant difference in female participants, particularly in reducing nausea and weakness [10]. The effect of NAC on electrolytes and markers of oxidative stress in alcohol hangover has not yet been evaluated [3].

The aim of this study was to evaluate the effect of NAC on serum levels of electrolytes, enzymes, oxidative stress biomarkers and symptoms of veisalgia in binge drinking.

## 2. Materials and Methods

This was a randomized, double-blind, placebo-controlled clinical study involving healthy volunteers. The study was approved by the Slovenian National Medical Ethics Committee under the application number 0120-178/2020/9.

### 2.1. Healthy Volunteers

The study was conducted in a students’ room designed for studying and socializing and under the supervision of sober researchers, physicians and registered nurses.

The participants were medical students, aged 19 to 27, who regularly attend social gatherings where alcohol is consumed and had already experienced the symptoms of hangover. The participants had to be healthy, not pregnant, and without any chronic diseases or therapies that could influence the results. They should not have been taking any medications. On the day of the study, they should not have smoked or taken any other psychoactive substances or over-the-counter medications. Only volunteers who had not consumed any alcohol in the previous 24 h were included. They signed informed consent.

Participants in the study who continued drinking after the study ended, took any other psychoactive substances, or took other measures that supposedly could alleviate hangover symptoms were excluded.

### 2.2. Study Protocol

**Figure 1:**
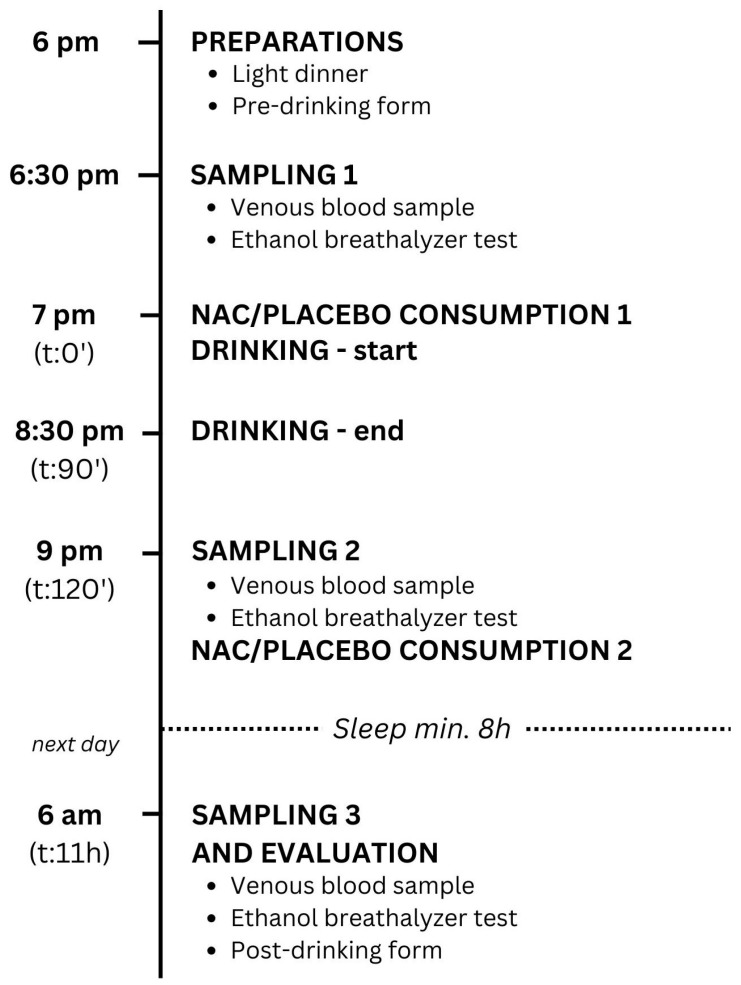
Study protocol. NAC—N-acetylcysteine, t—time after the start of drinking.

On the study day, the participants met the investigator at the study location at 6 pm. In the beginning, they all ate two slices of margherita pizza with approximately 250–350 calories and 5–8 g of fat each, so that none of them drank on an empty stomach. They filled out a pre-drinking evaluation form about veisalgia (score 1–10) and specific symptoms (score 1–10), as described in Section 2.4. Participants underwent a breath alcohol test using Dräger Alcotest 6820 to exclude volunteers who had been drinking before the study. The volunteers were weighed, their height was measured, and their BMI was calculated. The BMI is expressed in kg/m^2^, resulting from mass in kilograms and height in meters. The registered nurses took blood samples before drinking (Figure 1).

Each participant was assigned an identification number. Randomization was carried out using a computer. Based on their randomized numbers, volunteers were divided into two groups: one group received NAC with lemon juice (1.2 g NAC before and 1.2 g NAC after drinking alcohol), while the other group received a placebo (water mixed with lemon juice), as described in Section 2.3. The study began at 7 p.m., when they drank the contents of a numbered cup with NAC or placebo corresponding to their number. The researchers and volunteers on the scene were not aware which cups contained NAC or the placebo. Afterwards, they drank 40% *v*/*v* gin according to the participant’s preferences. The drinking was calm. They were mostly sitting and did not participate in any physical activity such as dancing. No other legal or illegal substances or medications were taken during the study (Figure 1).

The drinking ended at 8.30 p.m. The nurses took the second blood sample 30 min after a 1.5 h long drinking session. The volunteers also performed the second breath alcohol test (Figure 1).

At 9 p.m., they were given the second cup with 1.2 g of NAC or placebo corresponding to their randomized number. At the latest, one hour after the second blood sample was taken, all the participants went to sleep. They were instructed not to drink excessive amounts of water (Figure 1).

The next morning at 6 a.m. (9 h after drinking), the third blood sample was taken from all volunteers. They performed the third breath alcohol test and filled out a post-drinking form about veisalgia (score 1–10) and specific symptoms (score 1–10) (described in Section 2.4), including the Alcohol Hangover Severity Scale (AHSS) [2] (Figure 1).

### 2.3. N-Acetylcysteine and Placebo

The cups with pure-substance NAC (Fagron UK Ltd., Newcastle upon Tyne, UK) or placebo were prepared and numbered by the physician not attending the drinking session just before the study. NAC was dissolved in water and sweetened with concentrated lemon juice (5%) to mask the taste of NAC. The placebo cups contained water with dissolved concentrated lemon juice (5%). Dissolved NAC and placebo were in white, numbered cups without any other markings and had the same taste, color and smell. The NAC dosage was determined based on reports from users who experienced an improvement in hangover symptoms after using 1–2 doses of 1.2 g of NAC (1–2 doses of over-the-counter NAC, consisting of two 600 mg tablets each).

### 2.4. Pre- and Post-Drinking Evaluation of Hangover Severity

The severity of symptoms and veisalgia before drinking and the next morning after drinking were tested with a questionnaire in which the volunteers subjectively evaluated the severity of their veisalgia (score 1–10) and specific symptoms including wellbeing (10–1), headache (1–10) and thirst (1–10). They graded veisalgia and symptoms with 1 to 10 points, where 1 point meant that the symptom is absent and number 10 represented the worst severity of the symptom experienced in life. For sense of wellbeing, the scoring was reversed.

The next morning after drinking, the volunteers filled out a standardized questionnaire including the Alcohol Hangover Severity Scale (AHSS), which includes twelve symptoms correlating with veisalgia: palpitation, sweating, confusion, apathy, abdominal pain, shivering, dizziness, nausea, clumsiness, concentration problems, thirst and fatigue. Every symptom was graded on the scale from 0 to 10 points, where 0 points meant the absence of the symptom and 10 points meant the extreme severity of the symptom. The result of the AHSS questionnaire was calculated for every participant as the average number of points of all of the 12 symptoms [2].

### 2.5. Biochemical Blood and Breath Analysis

Serum levels of sodium, potassium, urea, creatinine, creatinine kinase, lactate dehydrogenase and liver enzymes (aspartate and alanine aminotransferase, gamma glutamyl transferase) and ethanol were measured at the Institute of Clinical Chemistry and Biochemistry, University Medical Centre, Ljubljana.

Serum concentrations of urea and creatinine, as well as the catalytic activity of creatinine kinase, lactate dehydrogenase and liver enzymes (aspartate and alanine aminotransferase, gamma glutamyl transferase), were measured spectrophotometrically (Advia 2400 analyzer and reagents; Siemens Healthcare, Erlangen, Germany). The measurements of the concentrations of sodium and potassium in serum were based on indirect potentiometric procedures using ion selective electrodes (Advia 2400 analyzer and reagents; Siemens Healthcare, Erlangen, Germany).

The concentration of ethanol in blood was measured using the enzymatic method with colorimetric detection (Vitros 350 analyzer and reagents; Ortho-Clinical Diagnostics, Rochester, NY, USA). Briefly, the enzyme alcohol dehydrogenase converts ethanol to acetaldehyde and reduces NAD^+^ to NADH (nicotinamide adenine dinucleotide), which is detected with the measurement of its absorbance at 340 nm.

The breath alcohol test was performed using a professional and calibrated Dräger Alcotest 6820, which employs an electrochemical sensor to measure blood alcohol content. This method is based on the oxidation of alcohol in the breath sample, producing an electrical current proportional to the alcohol concentration.

### 2.6. Quantification of 8-Hydroxydeoxyguanosine and N-Epsilon-Hexanoyl-Lysin in Blood Samples

To quantify the levels of 8-hydroxy-deoxyguanosine (8-OHdG) and hexanoyl-lysine (HEL) in blood samples, serum was collected without any additives and separated via centrifugation (at 1500× *g* for 10 min). Aliquots were then stored at −20 °C until analysis. All serum samples were analyzed together in a single batch. Competitive ELISA immunoassays (Japan Institute for the Control of Aging, Nikken SEIL Co., Ltd., Fukuroi, Japan) were utilized to measure 8-OHdG and HEL levels in serum samples. The detection limit was 0.5 µg/L for 8-OHdG and 2 nmol/L for HEL.

### 2.7. Statistical Analysis

The data are presented as means and standard deviations. Data were checked for normality using the Kolmogorov–Smirnov (K-S) test. The statistical significance of differences between the normally distributed laboratory values was evaluated by analysis of variance (ANOVA), followed by the Bonferroni post hoc test for multiple-group comparisons at the three points (before, immediately after, and the morning after drinking). Laboratory values that were not normally distributed were evaluated using non-parametric tests. The Kruskal–Wallis test was used to analyze the differences between groups in the laboratory results globally, and post hoc comparisons between groups were conducted using the Mann–Whitney test. Categorical values were analyzed using the Chi-square test. A *p*-value of 0.05 was considered significant. Analyses were carried out with IBM SPSS Statistics for Windows, Version 23.0., Armonk, NY, USA.

## 3. Results

Forty-five volunteers (aged 19–27 years), who were all students, were willing to participate in this study. They were randomized into an intervention group (23) and a placebo (22) group. However, five volunteers (two males) with an average age of 23.2 ± 1.6 years, a weight of 70.4 ± 11.0 kg, and a BMI of 23.2 ± 2.0 kg/m^2^ were excluded from the study due to their continued drinking after the study ended. Forty students (20 male), aged 23 ± 2 years, were included in the study. They were all healthy and not taking any medications. Additionally, on the day of the study, they did not consume any other psychoactive substances or over-the-counter medications. There were no significant differences between the included and excluded participants in terms of gender (*p* = 0.69), age (*p* = 0.81), weight (*p* = 092), or BMI (*p* = 0.77).

The NAC group consisted of 20 volunteers (10 male) with an average age of 23 ± 2 years and an average weight of 72.0 ± 13.8 kg and BMI of 23.0 ± 2.6 kg/m^2^. The placebo group also consisted of 20 volunteers (10 male) with an average age of 23 ± 2 years and an average weight of 71.8 ± 13.9 kg and BMI of 23.4 ± 2.7 kg/m^2^. There were no differences between the NAC and placebo groups in terms of gender (*p* = 1.00), age (*p* = 1.00), weight (*p* = 0.95) or BMI (*p* = 0.98).

During the study, the volunteers in both the NAC and placebo groups consumed 316 ± 76 mL (99.7 ± 24.0 g of ethanol) and 315 ± 80 mL (99.4 ± 25.2 g of ethanol) of gin, respectively, with no significant difference between the two groups (*p* = 0.84).

The breath alcohol levels in both the NAC and placebo groups were measured to be 0.53 ± 0.09 mg/L and 0.55 ± 0.10 mg/L, respectively, with no significant difference between the two groups (*p* = 0.50). Half an hour after drinking, the blood ethanol concentration in the NAC group was 1.40 ± 0.26 g/kg, while in the placebo group it was 1.44 ± 0.28 g/kg, with no significant difference observed between the groups (*p* = 0.65) (Table 1).

Symptoms and veisalgia were reported by both groups the morning after drinking (Table 1).

The severity of feeling bad, headache, and thirst the next morning was significantly worse in both the NAC (*p* = 0.01, *p* = 0.01, *p* = 0.01, respectively) and placebo groups (*p* = 0.01, *p* = 0.01, *p* = 0.01, respectively) compared to before drinking. However, there was no significant difference in the severity of feeling bad, headache, and thirst between the NAC and placebo groups the next morning (*p* = 0.38, *p* = 0.96, *p* = 0.89, respectively). The severity of veisalgia (scored on a scale of 1–10) was also worse the next morning compared to before drinking in both the NAC (*p* = 0.01) and placebo groups (*p* = 0.01), but there was no significant difference in the severity of veisalgia between the NAC and placebo groups the next morning (3.4 ± 2.1 and 3.7 ± 2.7, respectively) (*p* = 0.63) (Table 1).

The severity of veisalgia, rated on the Alcohol Hangover Severity Scale of 0 to 10, was found to be similar between the NAC and placebo groups the following morning, with scores of 2.5 ± 1.3 and 2.6 ± 1.1, respectively (*p* = 0.53).

The serum sodium level was increased after drinking in both the NAC group (143.0 ± 2.5 mmol/L vs. 140.7 ± 2.3 mmol/L, *p* = 0.01) and the placebo group (143.1 ± 1.8 mmol/L vs. 140.1 ± 1.5 mmol/L, *p* = 0.01) compared to the level before drinking. NAC had no effect on the sodium level after drinking (*p* = 0.88) (Table 1).

The next morning, the serum sodium level had returned to its pre-drinking level in both the NAC group (140.3 ± 1.6 mmol/L, *p* = 1.00) and the placebo group (141.3 ± 1.7 mmol/L, *p* = 0.10). NAC did not have a significant effect on the sodium level the next morning (*p* = 0.06) (Table 1).

The serum urea level was found to be decreased in both NAC and placebo groups the next morning after drinking compared to its levels before drinking (*p* = 0.01 and *p* = 0.01, respectively) (Table 1). Although serum urea after drinking was found to be between its levels before drinking and the next morning, it was not significantly different compared to its levels before drinking and the next morning in either group (Table 1).

NAC had no effect on urea levels after drinking (*p* = 0.47) or the next morning (*p* = 0.58) (Table 1).

The serum level of oxidative biomarker 8-OHdG was increased after drinking in both NAC (56.5 ± 51.8 µg/L vs. 4.3 ± 1.7 µg/L, *p* = 0.01) and placebo groups (38.4 ± 25.2 vs. 3.9 ± 1.3 µg/L, *p* = 0.01) compared to its levels before drinking. The next morning, the levels were similar to those after drinking in both NAC (53.3 ± 50.0 µg/L, *p* = 0.65) and placebo groups (41.1 ± 24.4 µg/L, *p* = 0.78).

NAC had no effect on 8-OHdG level after drinking (*p* = 0.71) or the next morning (*p* = 0.38) (Table 1).

Neither alcohol nor NAC had any effect on creatinine, liver and muscle enzymes, or HEL.

## 4. Discussion

In this randomized, double-blind, placebo-controlled study, binge alcohol drinking led to an increase in serum sodium and DNA oxidative marker (8-OHdG) levels at the end of the drinking session. NAC had no effect on serum sodium and 8-OHdG levels at the end of the drinking session.

The morning after drinking, volunteers experienced symptoms of veisalgia, along with decreased serum urea and increased serum 8-OHdG levels, while the levels of sodium had returned to its pre-drinking levels. NAC had no impact on either the laboratory results or the symptoms of veisalgia the following morning. This is consistent with findings in mildly intoxicated volunteers, where NAC did not reduce symptoms [10], but it is not consistent with animal models, where NAC decreased behavioral changes [11]. The ineffectiveness of NAC in its failure to improve subjectively assessed symptoms the next morning may be due to the insufficient dosing of NAC regarding severe intoxication. In the study where participants had only 0.5 permille of alcohol in their blood and took a comparable dose of NAC (600–1800 mg), NAC reduced nausea and weakness in female participants [10]. In our study, the participants had an almost three-fold higher blood alcohol level but took almost the same dose of NAC. 

The etiology of veisalgia is likely to be multifactorial, and this study provides some additional insight into this as well. 

The first possible mechanism of veisalgia observed in this study was an increase in serum sodium concentration, likely due to alcohol induced diuresis and dehydration. However, it is noteworthy that the serum sodium concentrations remained within the reference range in volunteers with 1.4 permille, despite the fact that hypernatremia is not uncommon in acute alcohol intoxication [12]. Dehydration caused by ethanol is commonly cited as a significant contributor to veisalgia as well, given that ethanol is a well-known diuretic. Alcohol initially increases diuresis by decreasing vasopressin secretion or/and sensitivity to vasopressin, but over a longer period of time, such as towards morning, there is an increase in vasopressin secretion, leading to water retention [13,14,15,16]. In this study, we observed a corresponding sequence where the levels of sodium first increased and then decreased to pre-drinking levels along with a decrease in urea levels. This could be due to water retention in the morning, as the volunteers did not consume excessive amounts of water. The beneficial effect of hydration in veisalgia could be evaluated in future studies of veisalgia, along with NAC, as decreased serum urea concentration after alcohol consumption has also been observed in similar studies [12,17]. 

The second observed mechanism that could contribute to veisalgia in this study was oxidative stress. The serum oxidative stress marker, 8-OH-dG, increased after drinking and remained elevated until the following morning. Oxidative stress due to ethanol poisoning has already been identified through animal models [18,19,20], as well as in humans, where elevated markers of oxidative stress and decreased levels of antioxidants (glutathione and superoxide dismutase) were measured during alcohol consumption [21]. In this study, the antioxidant NAC had no impact on the markers of oxidative stress, despite it being shown to reduce the markers of oxidative stress in an animal model of alcohol exposure [11]. This could be due to the inadequate dosing of NAC, which is already described as a limitation of this study. Another potential factor to consider is the use of lemon juice in both the NAC and placebo groups. As lemon juice is known for its antioxidant properties, attributed to its vitamin C and limonoid content, it could influence the results. As no significant adverse side effects of NAC were observed in this study or similar studies [22], a higher dose of NAC without use of lemon juice could be used in future studies on the prevention of oxidative stress and veisalgia.

Further research also seems warranted because there is a reasonable explanation for the potential positive effects of NAC on the mechanisms underlying alcohol hangover. Specifically, NAC replenishes antioxidant glutathione levels, which are depleted during ethanol intoxication [6], and can possibly reduce oxidative stress. Glutathione and its product, cysteine-glycine, can also form adducts with acetaldehyde [7,8,9]. A potential second mechanism involves the regeneration of glutathione by glutathione reductase, which may lead to an indirect increase in NAD^+^ levels via the pentose phosphate pathway, tricarboxylic acid cycle, and glycolysis. The increased availability of NAD^+^ could boost the activity of acetaldehyde dehydrogenase and facilitate the breakdown of acetaldehyde into acetate [23].

## 5. Conclusions

Binge drinking causes a transient increase in serum sodium and a prolonged increase in oxidative marker 8-OHdG levels. NAC had no effect on sodium and 8-OHdG levels. 

## Figures and Tables

**Table 1:** The laboratory results before, after, and the morning following alcohol drinking in both NAC and placebo groups.

Parameter (Units; Reference Range)	NAC			Placebo		
	Before Drinking	After Drinking	Next Morning	Before Drinking	After Drinking	Next Morning
Blood ethanol (g/kg)	0.00 ± 0.00 *	1.40 ± 0.27 *,#	0.01 ± 0.03 #	0.00 ± 0.00 *	1.44 ± 0.28 *,#	0.03 ± 0.08 #
Sodium (mmol/L; 135–145)	140.7 ± 2.3 *	143.0 ± 2.5 *,#	140.3 ± 1.6 #	140.1 ± 1.5 *	143.1 ± 1.8 *,#	141.3 ± 1.7 #
Potassium (mmol/L; 3.8–5.5)	4.2 ± 0.5 *	3.8 ± 0.4 *,#	4.3 ± 0.3 #	4.2 ± 0.4 *	3.9 ± 0.4 *,#	4.2 ± 0.3 * #
Urea (mmol/L; 3.2–7.4)	5.4 ± 1.0 †	5.0 ± 1.0	4.5 ± 0.8 †	5.3 ± 1.2 †	4.8 ± 1.1	4.4 ± 0.8 †
Creatine (μmol/L; 64–100)	83.2 ± 18.3	78.3 ± 14.8	75.7 ± 10.8	77.8 ± 11.7	75.8 ± 11.3	75.0 ± 10.1
oGF (mL/min)	88.7 ± 5.8	89.5 ± 2.5	90.0 ± 0.0	90.0 ± 0.0	90.0 ± 0.0	90.0 ± 0.0
AST (μkat/L; <0.58)	0.28 ± 0.06	0.27 ± 0.07	0.30 ± 0.07	0.31 ± 0.08	0.30 ± 0.09	0.34 ± 0.08
ALT (μkat/L; <0.10)	0.38 ± 0.17	0.38 ± 0.19	0.39 ± 0.18	0.39 ± 0.25	0.40 ± 0.26	0.42 ± 0.25
gGT (μkat/L; <0.92)	0.25 ± 0.11	0.25 ± 0.11	0.25 ± 0.11	0.25 ± 0.11	0.25 ± 0.11	0.24 ± 0.11
CK (μkat/L; <2.85)	2.13 ± 0.97	2.17 ± 0.95	2.04 ± 0.80	2.58 ± 1.74	2.62 ± 1.70	2.36 ± 1.50
LDH (μkat/L; <4.13)	2.80 ± 0.33	2.93 ± 0.48	2.87 ± 0.39	2.95 ± 0.35	2.92 ± 0.32	2.84 ± 0.32
HEL (nmol/L)	145.7 ± 65.2	191.1 ± 181.3	210.7 ± 246.6	162.6 ± 83.8	317.4 ± 487.6	245.6 ± 336.0
8-OH-dG (µg/L)	4.3 ± 1.7 *,†	56.5 ± 51.8 *	53.3 ± 50.0 †	3.9 ± 1.3 *,†	38.4 ± 25.2 *	41.1 ± 24.4 †
Wellbeing (10–1)	7.8 ± 1.2 †		6.6 ± 1.5 †	8.1 ± 0.9 †		6.6 ± 1.9 †
Headache (1–10)	1.0 ± 0.0 †		2.4 ± 1.7 †	1.0 ± 0.0 †		2.6 ± 2.1 †
Thirst (1–10)	1.2 ± 0.5 †		4.7 ± 2.2 †	1.3 ± 0.6 †		5.4 ± 2.3 †
Veisalgia (1–10)	1.1 ± 0.3 †		3.4 ± 2.1 †	1.1 ± 0.3 †		3.7 ± 2.7 †
AHSS (0–10) [4]			2.5 ± 1.3			2.6 ± 1.1

Legend: CK—creatinine kinase; LDH—lactate dehydrogenase; AST—aspartate aminotransferase; ALT—alanine aminotransferase; gGT—gamma glutamyl transferase; 8-OHdG—8-Hydroxydeoxyguanosine; HEL—N-epsilon-hexanoyl-lysine, HEL; AHSS—Alcohol Hangover Severity Scale; μkat/L = microkatal per liter; *—statistically significant difference between the results at the beginning of drinking and at the end of drinking alcohol; †—statistically significant difference between the results at the beginning of drinking and the next day after drinking alcohol; #—statistically significant difference between the results at the end of drinking and the next day after drinking alcohol; *p* < 0.05. The data are presented as means and standard deviations.

## Data Availability

Data are contained within the article.
